# Effects of Caloric Restriction on DNA Damage: A Comparison of Very Low-Calorie and Standard Reduced-Calorie Diets in Obesity—Non-Randomised, Quasi-Experimental Clinical Intervention Study

**DOI:** 10.3390/nu18121985

**Published:** 2026-06-19

**Authors:** Mirta Milić, Ivan Ožvald, Alice Mannocci, Stefano Bonassi, Hrvoje Radašević, Maja Nikolić, Dragan Božičević, Lidija Duh, Martina Matovinović, Martina Bituh

**Affiliations:** 1 Division of Toxicology, Institute for Medical Research and Occupational Health (IMROH), 10001 Zagreb, Croatia; mnikolic@imi.hr; 2 Neuropsychiatric Hospital Dr. Ivan Barbot of Popovača, 44317 Popovača, Croatia; ivan.ozvald@bolnicapopovaca.hr; 3 Department for the Promotion of Human Sciences and Quality of Life, University San Raffaele, 00166 Rome, Italy; alice.mannocci@uniroma5.it; 4 Clinical and Molecular Epidemiology, IRCCS San Raffaele Roma, 00166 Rome, Italy; 5 Andrija Štampar Teaching Institute of Public Health, 10000 Zagreb, Croatia; hrvoje.radasevic@stampar.hr; 6 Special Hospital for Extended Treatment of Duga Resa, 47250 Duga Resa, Croatia; bozicevicdragan@gmail.com (D.B.); lidijaduh1@gmail.com (L.D.); 7 Department of Internal Medicine, University Hospital Centre Zagreb, 10000 Zagreb, Croatia; martina_10000@yahoo.com; 8 Faculty of Kinesiology, University of Zagreb, 10000 Zagreb, Croatia; 9 Laboratory for Food Chemistry and Biochemistry, Faculty of Food Technology and Biotechnology, University of Zagreb, 10000 Zagreb, Croatia; martina.bituh@pbf.hr

**Keywords:** VLCD, standard reducing diet, comet assay, micronucleus *cytome* assay

## Abstract

**Background:** Obesity is a chronic endocrine–metabolic disorder. The risk of comorbidities increases with a higher body mass index (BMI), particularly when BMI ≥ 35.0 kg/m^2^. Common complications include insulin resistance, type 2 diabetes, dyslipidemia, and chronic low-grade inflammation, which collectively impair DNA stability by promoting the formation of genotoxic species. **Methods:** This non-randomised, quasi-experimental clinical intervention study included 53 participants (both sexes) with a BMI ≥ 35.0 kg/m^2^, who were assigned to parallel experimental or control streams based on clinical needs and institutional eligibility. During a three-week intervention, the experimental group received a hospital-supervised very-low-calorie diet (VLCD; ~600 kcal/day) under continuous medical monitoring. Conversely, the control group followed a standard reduced-calorie diet (SRD) of 1500 kcal/day in a free-living home environment. Before and after the intervention, primary, oxidative, and permanent DNA damage were measured using alkaline, FPG-modified comet (peripheral blood mononuclear cells), and cytokinesis-block micronucleus *cytome* assays (phytohaemagglutinin-stimulated binucleated lymphocytes), alongside anthropometric and biochemical tracking. **Results:** Within-group evaluations revealed that both dietary regimens improved several metabolic health indicators, notably modulating insulin resistance, lipid profiles, and leukocyte counts. However, participants in the VLCD stream experienced significantly greater downward changes in body weight, BMI, and absolute lipid values. Crucially, the VLCD intervention was associated with a highly significant within-group reduction in parameters of permanent chromosomal damage, effectively halving the frequencies of micronuclei and nuclear buds, independent of baseline variations, in adjusted multivariate regression models. Conversely, the home-based SRD regimen demonstrated no measurable impact on permanent genomic damage. Neither diet induced a significant change in repairable primary or oxidative DNA lesions over this short timeframe. **Conclusions:** These exploratory findings suggest that strict calorie restriction can rapidly stabilise genome stability in advanced clinical settings, warranting future randomised controlled trials with long-term longitudinal follow-up to assess permanent risk reductions. Due to structural baseline variations in age, chronic comorbidities, and compliance environments between the cohorts, direct comparative superiority cannot be definitively established.

## 1. Introduction

Obesity is a chronic disease characterised by excessive fat accumulation that impairs health and is classified under ICD-11 code 5B81 [1]. Since direct assessment of body fat is often impractical, obesity is typically defined using the body mass index (BMI) [2,3]. According to the World Health Organisation (WHO), the global prevalence of obesity has more than doubled since 1990 [4]. According to Eurostat 2022, overweight and obesity affected 57.2% and 16.5% of adults in Croatia [5].

Obesity is a significant risk factor for metabolic complications, including hyperglycemia, dyslipidemia, and chronic inflammation, which collectively promote genomic instability [6,7]. DNA damage in obesity arises from multiple mechanisms, including base oxidation, alkylation, methylation, nitration, deamination, strand breaks, cross-linking, and adduct formation [8,9]. Indeed, overweight and obese individuals exhibit greater DNA damage than normal-weight individuals [10,11,12] and lower or even inactive DNA repair mechanisms activity [13].

Diet plays a central role in DNA integrity. Genotoxic effects may occur directly through DNA interactions, indirectly via inadequate intake of nutrients essential for DNA synthesis and repair, or through disruption of redox balance and induction of oxidative stress [14,15,16]. Excessive caloric intake promotes the generation of reactive oxygen species (ROS), thereby stimulating inflammatory pathways [17]. In contrast, caloric restriction supports DNA repair mechanisms, regardless of the type of damage [17].

Reducing energy intake remains the cornerstone of obesity management, with interventions tailored to BMI and comorbidities [18,19,20,21,22]. Very-low-calorie diets [VLCDs; <800 kcal/day) are recommended for individuals with a BMI ≥ 27 kg/m^2^ and associated comorbidities, resulting in rapid weight loss, reduction in fat mass, and metabolic improvements [23,24,25,26,27]. However, adherence to such severely calorie-restricted diets is generally lower, making compliance more challenging [28].

In Croatia, the standard reducing diet (SRD; 1200–1700 kcal/day) is widely used in hospital settings, with a macronutrient composition of 20–25% protein, 50–55% carbohydrates, and ≤30% fat.

The data from the three-week VLCD indicate improved genome stability [29,30]. Research on the impact of weight loss on DNA stability in individuals with BMI ≥ 35 kg/m^2^ remains limited and yields inconclusive results. This study aims to evaluate and compare the effects of a VLCD of approximately 600 kcal with an SRD of about 1500 kcal on DNA damage, including primary, oxidative, and permanent damage, using comet and micronucleus *cytome* assays over a three-week period. Additionally, it will assess changes in anthropometric and biochemical parameters in this population group. We hypothesise that VLCD will lead to greater genomic stability and more substantial reductions in obesity-related anthropometric and biochemical parameters than SRD.

## 2. Materials and Methods

**Figure 1:**
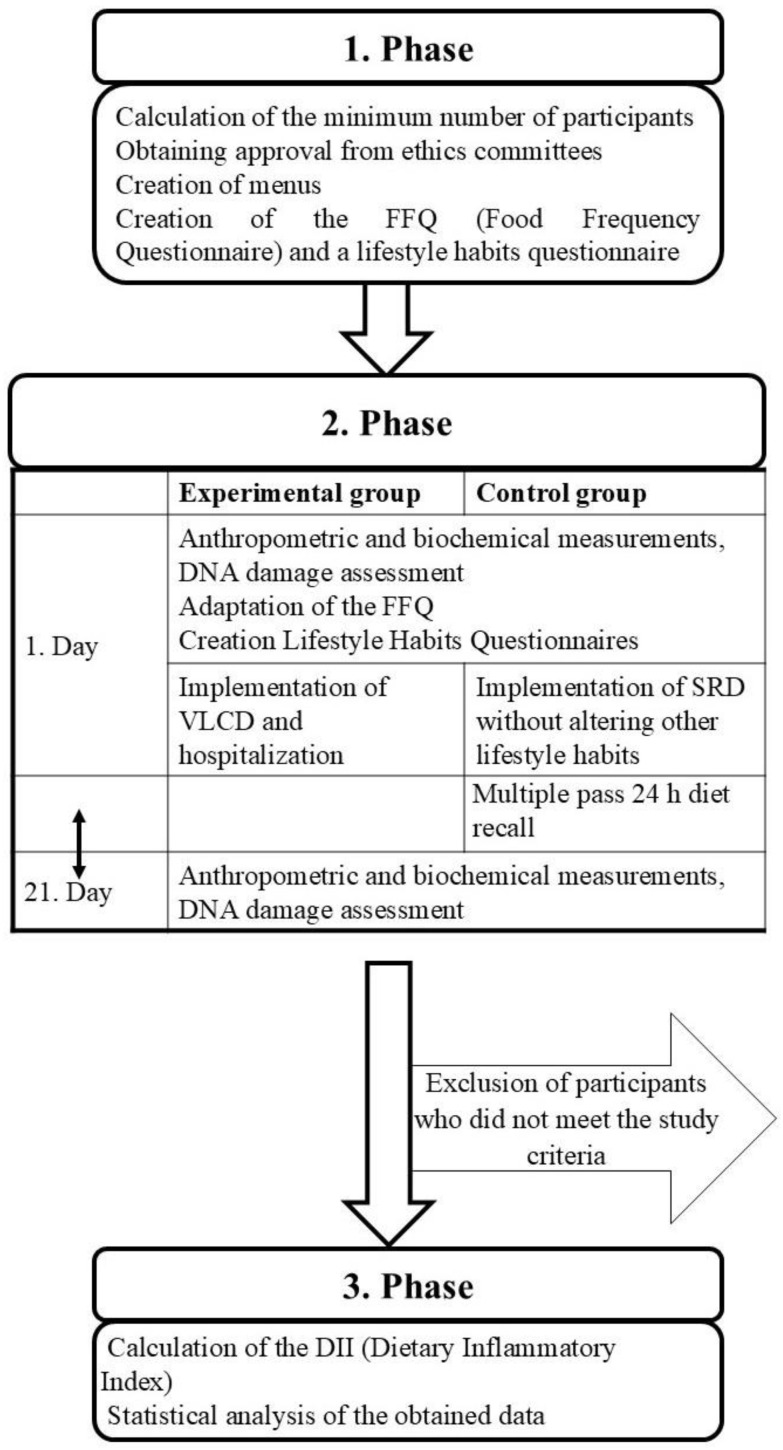
Study design. Overview of the experimental protocol, VLCD—Very Low-Calorie Diet, SRD—Standard Reduced Diet.

The non-randomised, quasi-experimental clinical intervention study was structured into three distinct phases, as illustrated in Figure 1. The initial phase focused on determining the minimum number of participants needed to validate the research hypothesis. This calculation was based on 5% significance level (α < 0.05), a study power (β = 0.2), and the effect size derived from Soares et al. (2016) [31]. We used SPSS software (SPSS Inc. 2004 for Windows, version 13.0. Chicago, IL: SPSS Inc., USA), and as in the [31] and other previous studies and clinical relevance, the effect expected was the minimal difference that should be detected in weight loss, DNA damage in comet assay and biochemical blood markers after the diet, important for cardiometabolic risk factors that we also used in our study when the dietary intervention was minimal, such as 500 kcal reduction. The result indicated a requirement of 52 participants, evenly divided between the VLCD and SRD groups. In preparation for the study, 21 SRD menus were crafted.

Additionally, consent forms were developed for participant enrollment, and food frequency questionnaires (FFQs) and a lifestyle habits questionnaire were designed.

The research was conducted in strict compliance with the Helsinki Declaration and received ethical clearance from multiple committees: The Special Hospital for Extended Treatment Duga Resa (approval No.: 08-08-970/19, 15 April 2019 and 18 October 2021), the Institute for Medical Research and Occupational Health in Zagreb (approval No.: 100-21/19-10, 6 June 2019), and the School of Medicine, University of Zagreb (approval No.: 380-59-10106-20-111/174; class: 641-01/20-02/01, 14 December 2020). Additionally, the study is registered under ClinicalTrials.gov identifiers NCT05055154 (registered on 14 June 2019) and NCT05007171 (registered on 10 August 2021). Participants were thoroughly briefed about the research objectives and procedures and provided informed consent for their voluntary participation.

The second phase commenced with participants signing informed consent forms, followed by initial measurements of anthropometric and biochemical parameters, as well as DNA damage indicators. During this phase, the nutritional intervention was implemented. Participants completed the FFQ and a questionnaire that assessed their lifestyle habits. Adherence in the SRD control group was monitored through unannounced 24 h dietary recalls. After three weeks of nutritional intervention, anthropometric, biochemical, and DNA damage parameters were measured again.

In the third phase, all collected data were entered into an Excel spreadsheet for statistical analysis, encompassing all measured and calculated parameters from both the experimental and control groups, which were subsequently compared to conclude the study.

### 2.1. Participants

The study involved 53 participants, representing both genders, with a BMI ≥ 35 kg/m^2^ and with secondary, acquired polygenic Class III obesity primarily driven by long-term lifestyle factors, positive energy balance, and sedentary behaviours. Patients presenting with diagnosed congenital or monogenic forms of obesity (e.g., Prader–Willi syndrome, melanocortin 4 receptor mutations) or obesity secondary to underlying neuroendocrine disorders were not present in the clinic sample, ensuring homogeneity in lifestyle-driven metabolic etiologies. Participants who were willing (and able, since all our participants were working in their regular jobs) to remain in the hospital under surveillance were selected for the VLCD group (experimental). Inclusion criteria for all participants included: BMI ≥ 35 kg/m^2^, stable body weight over the past three months, completion of at least three weeks of participation—including anthropometric measurements and venous blood sampling at both the start and end of the study—signed informed consent, completion of questionnaires on lifestyle habits and health status, and the FFQ. Exclusion criteria comprised pregnancy, being a minor, the presence of acute or neoplastic diseases, recent surgical procedures (with or without local or general anaesthesia) within six months before participation, and recent diagnostic procedures involving ionising or non-ionising radiation within six months before the study.

This study utilises a non-randomised, quasi-experimental clinical intervention design. Because participants were assigned to the parallel dietary streams based on their immediate clinical needs and willingness/eligibility for inpatient hospitalisation, severe selection bias is inherently present. The cohorts possess distinct motivation profiles and clinical histories that must be considered when evaluating comparative outcomes.

### 2.2. Experimental (VLCD) Group

Participants in the experimental group were also selected by a specialist in internal medicine and a subspecialist in diabetology and endocrinology from among patients who had not achieved weight loss through outpatient treatment and who agreed to undergo hospital-based obesity management. An additional exclusion criterion for this group was the risk of developing malnutrition during obesity therapy. The hospital treatment for obesity involved VLCD administration and meals prepared in the hospital kitchen. Due to potential health risks, participants were under continuous 24 h medical supervision throughout the intervention.

### 2.3. Control (SRD) Group

Participants in the control group were recruited via acquaintances, personal contacts, medical doctors and public social media posts. They followed an SRD at home at their own expense, without any additional lifestyle modifications or recommendations. An extra inclusion criterion for this group was their consent to be contacted twice during the study via the provided telephone number to assess food and beverage intake over the previous two days, with one of these assessments occurring on a weekend.

### 2.4. Anthropometric Measurements

Participants’ heights were measured using a stadiometer, and other anthropometric parameters were assessed with the InBody 270 scale (Cerritos, CA, USA). This included measurements of muscle and fat tissue mass, as well as the calculation of fat tissue percentage and BMI. The scale utilises bioelectrical impedance analysis with two different frequencies (20 kHz and 100 kHz) across five body segments (right and left arms, trunk, and right and left legs) to determine anthropometric parameters.

### 2.5. Biochemical Measurements

Blood sampling was conducted in accordance with established guidelines for venous whole blood collection [32]. Samples were collected at the Special Hospital for Extended Treatment Duga Resa and the former Unit for Mutagenesis (now Division of Toxicology) at the Institute for Medical Research and Occupational Health. Regardless of location, peripheral whole blood was drawn into single-use vacuum tubes supplied by BD (Becton, Dickinson and Company, Franklin Lakes, NJ, USA). A total of five tubes were collected: two with K_3_EDTA anticoagulant, two with coagulation activator, and one with Li-heparin anticoagulant. Immediately after collection, samples were transported upright in a portable cooler maintained at 4 °C, protected from light, and within the stability period relevant to each parameter, between the two facilities.

The blood tubes were allocated as follows: the K_3_EDTA tubes for complete blood count (CBC) and the assessment of DNA damage from peripheral blood mononuclear cells using alkaline and formamidopyrimidine DNA glycosylase (FPG) modified comet assay; the coagulation activator tubes for biochemical parameter analysis; and the Li-heparin tubes for the evaluation of chromosomal damage in phytohaemagglutinin-stimulated binucleated lymphocytes after 72 h culture, using the cytochalasin B-blocked micronucleus (MN) *cytome* assay (CBMN).

CBC and biochemical analyses were performed at the Special Hospital for Extended Treatment Duga Resa. CBC was measured immediately upon receipt of the sample using the Sysmex XS-1000i analyser (Kobe, Japan) with manufacturer-provided reagents. Selected biochemical parameters—including triglycerides, HDL cholesterol, glucose, and high-sensitivity C-reactive protein (hsCRP)—were determined using the Beckman Coulter AU 480 analyser (Brea, CA, USA) with Beckman Coulter reagents. LDL-cholesterol was calculated using the Friedewald equation [33]. Insulin levels were measured using a chemiluminescent microparticle immunoassay on the Architect i1000SR analyser (Abbott, IL, USA) with Abbott reagents.

### 2.6. DNA Damage Measurements

The alkaline, FPG comet and CBMN assays were performed at the (former) Mutagenesis Unit, now Division of Toxicology, Institute for Medical Research and Occupational Health in Zagreb, to assess DNA damage. The alkaline and FPG comet assay procedures were previously described [34] and are also available in the compendium of protocols [35]. For the comet assays, we used Tail Intensity (% DNA in the comet tail; damaged cells or their DNA after electrophoresis have a comet-like shape). Details of the CBMN are provided in our earlier publication [30]. MN is the biomarker of the breakage or loss of the chromosome, nuclear bud (NBUD)—of amplified genes, and sometimes even the beginning of the MN creation, nucleoplasmic bridge (NPB)—of dicentric chromosomes. Apoptotic cells are those undergoing programmed cell death due to excessive unrepaired DNA damage, and necrotic cells are those that are naturally dying. Mitotic index shows whether the cell division is slower than average values around 2 (according to the frequency of mononucleated, binucleated, tri and tetra nucleated cells after the first division and repair in that division and adding the cytochalasin B to stop the cytokinesis to check for the unrepaired DNA damage remained in the shared cytoplasm of the two-daughter nucleus) [36,37].

### 2.7. Nutrition Intervention

The VLCD used in this study was developed at the Special Hospital for Extended Obesity Treatment. The daily menu provided approximately 600 kcal, with macronutrient distribution comprising 50–60% complex carbohydrates with a low glycemic index, 20–25% protein, and 25–30% fat. Participants consumed their food in three meals, accompanied by larger quantities of unsweetened fluids, primarily water. Due to space constraints in the hospital, patients did not participate simultaneously; however, their meal plans and the entire research protocol were standardised. These meals served as the sole food source, as patients were under constant medical supervision, with no cafeteria or external food services available and with visits limited.

For the control group, a 1500 kcal reduction diet (SRD) was developed using the “Prehrana” computer program (Infosistem d.d., Zagreb, Croatia). The control diet was designed to be diverse, nutritionally balanced, and aligned with Croatian hospital nutrition standards. It comprised approximately 50–55% carbohydrates, 20–25% protein, and ≤30% fat, with a total daily energy intake of around 1500 kcal. Meals were distributed in five servings, including carbohydrate sources such as fruits, vegetables, grains, and legumes; protein mainly from poultry, fish, legumes, and lean veal; and fats from plant oils, including olive, sunflower, and pumpkin seed oils. Food preparation involved boiling, stewing, and baking with minimal fat, and ensuring at least two cooked meals were served daily. Participants were encouraged to drink about 2 litres of fluids, mainly unsweetened teas and water. The diet included over 500 g of fruits and vegetables daily to promote increased fibre intake.

Fat from different sources, such as plant oils and animal fats, is metabolised differently in the body. The ratio of saturated fatty acids (typically found in animal fats) to monounsaturated/polyunsaturated fatty acids (typically found in plant oils) strongly influences cellular membrane integrity, mitochondrial function, and downstream oxidative DNA damage [38]. For the SRD cohort, the dietary fat component (≤30% of total daily energy) was deliberately balanced to achieve a 2:1 ratio of plant-derived oils (primarily mono- and polyunsaturated fats from olive, pumpkin seed, and sunflower oils) to animal-derived fats. In the VLCD group, fat intake was restricted to approximately 16–20 g/day, with a focus on plant-derived lipids to supply essential fatty acids, while keeping saturated animal fats to a minimum to prevent lipid-induced oxidative stress.

Before the nutritional intervention, dietary habits were assessed using a modified FFQ based on Mulligan et al. (2014) [39]. Data from the FFQ were analysed with the FETA (FFQ EPIC Tool for Analysis), version 2.53 for Windows, last updated 15 March 2013, available online for download: https://www.epi.ims.cam.ac.uk/research/) (accessed on 1 April 2019). 

Using FFQ data, the Dietary Inflammatory Index (DII) was calculated to assess the inflammatory potential of participants’ diets before the intervention. For the experimental group, the DII was based on the average of the three-week VLCD, while for the control group, it was calculated from a 24 h dietary recall. The DII methodology follows Shivappa et al. (2014) [40], considering 45 dietary components—nine with pro-inflammatory effects (energy, protein, total fat, carbohydrates, saturated fatty acids, trans fats, cholesterol, iron, and vitamin B12) and 36 with anti-inflammatory effects (monounsaturated and polyunsaturated fatty acids, ω-3 and ω-6 fatty acids, dietary fiber, alcohol, vitamins A, D, E, C, B6, β-carotene, thiamine, riboflavin, niacin, folic acid, magnesium, selenium, zinc, flavan-3-ols, flavones, flavonols, flavanones, anthocyanidins, isoflavones, caffeine, garlic, onion, pepper, oregano, rosemary, eugenol, saffron, ginger, turmeric). The food and beverage composition data were obtained from databases of food chemical composition [41,42,43]. Retention factors were applied to flavonoids affected by different cooking methods: 0.59 for frying, 0.50 for baking, and 1.09 for boiling, to account for nutrient loss or retention during preparation [44]. Using Shivappa’s published methodology to calculate DII scores separately for each dietary assessment for specific pro- and anti-inflammatory nutrients and foods, and to convert them to standardised values (z-scores), two dietary assessment methods yielded comparable DII scores.

It must be emphasised that the DII was modelled using different dietary-tracking granularities across the streams—prospective 3-week menu averages for the VLCD group versus retrospective 24 h recalls for the SRD control—thereby introducing a measurement bias that limits direct cross-comparisons between the groups’ inflammatory indices.

### 2.8. Data Analysis

All statistical analyses were performed using SPSS software, version 13.0 for Windows (SPSS Inc., Chicago, IL, USA).

Descriptive statistics were computed for all variables. Continuous variables were summarised as medians and interquartile ranges, while categorical variables were expressed as absolute and relative frequencies. Data distribution was checked using visual inspection and the Shapiro–Wilk test to determine the appropriate statistical approach.

Within-group comparisons (pre-T0 vs. post-intervention T1) were assessed using the Wilcoxon signed-rank test. In contrast, between-group differences (VLCD vs. SRD) at each time point were evaluated with the Mann–Whitney U test for continuous variables and the χ^2^ test or Fisher’s exact test for categorical variables.

Correlations between changes (Δ) in the Dietary Inflammatory Index (ΔDII) and in DNA damage parameters (ΔTail Intensity Mean, Δfreq MN, Δfreq NBUD, ΔMN + NBUD, ΔNPB, Δapoptotic cells, Δnecrotic cells, and ΔFPG) were explored using Spearman’s correlation coefficients. Δ represents the change from baseline to follow-up (T1 − T0).

Multivariate regression analyses were conducted using the DNA damage parameters measured at T1 (Tail Intensity Mean, FPG, freq MN, freq NBUD, MN + NBUD, NPB, apoptotic cells, and necrotic cells) as dependent variables.

For continuous outcomes without zero values, linear regression models were fitted using both the original and natural-log transformations of the dependent variable, and the two model forms were compared to determine the best fit. Model selection was based on goodness-of-fit indices (R^2^) and residual diagnostics, including normality and homoscedasticity. The model showing more symmetric residuals and constant variance was retained for the final analyses.

For outcomes with a limited number of zero values (≤10% of the sample), a logarithmic transformation was applied after adding a constant of 0.01 to allow computation of ln(Y + 0.01), as recommended by Osborne [45]. For outcomes with a high proportion of zeros (e.g., necrotic cells, >70%), logistic regression models were used to account for the zero-inflated distribution.

Two types of models were estimated:

- Model 1 (basic adjustment): included the baseline value of the dependent variable and the diet group (VLCD vs. SRD = 0).
- Model 2 (extended adjustment): included the same variables as Model 1 plus the covariates showing an association with the dependent variable at *p* < 0.20 in univariate analyses. If more than five covariates met this criterion, only those with the lowest *p*-values were retained, maintaining an approximate ratio of one covariate per ten observations [46,47]. All statistical tests were two-tailed, and a *p*-value < 0.05 was considered statistically significant.

## 3. Results

### 3.1. Participant Flow and Baseline Characteristics

A total of 61 people with obesity participated in the study, of whom 53 (87%) fully adhered to their dietary plans. Among the 32 participants in the experimental group undergoing hospital treatment for obesity, complete data were collected for 26 (81.3%). Four participants discontinued hospital treatment for obesity during the study. One participant was discharged before completing the treatment/study due to a SARS-CoV-2 outbreak at the Special Hospital for Extended Treatment Duga Resa. Additionally, one participant’s samples were not analysed at the Institute for Medical Research and Occupational Health due to epidemiological measures to control the outbreak. Of the 29 participants in the control group who completed the survey, 27 (93%) met all the study criteria. One participant was excluded because his BMI at the start of the study was below 35 kg/m^2^ (although we initially reported it as 35.7 kg/m^2^), and another participant did not attend the anthropometric measurements and blood sampling three weeks after the study commenced.

### 3.2. Demographic and Health Characteristics of the VLCD Group

Detailed demographic and clinical data are summarised in Table 1. Out of the 26 participants who completed the study, an equal number were women (13, 50%) and men (13, 50%). The median age was 58.5 years, with an interquartile range of 51.3 to 60.0 years. A history of tumours was reported by 8 participants (31%), while the remaining 18 participants (69%) had no diagnosed tumour disease at the time of the study. Most participants, 24 (92%), reported having multiple chronic diseases or comorbidities associated with obesity, whereas only 2 (8%) reported no chronic illnesses aside from obesity.

The most common comorbidities included hypertension, reported by 23 participants (88%); type 2 diabetes, reported by 19 participants (66%); and dyslipidemia, reported by 18 participants (69%). Regarding lifestyle habits, 24 participants (92%) identified as non-smokers, 2 (8%) as smokers. The majority (16 participants, 62%) led sedentary lifestyles, while 10 participants (38%) engaged in regular physical activity, primarily through walking and household chores.

### 3.3. Demographic and Health Characteristics of the SRD Group

Detailed demographic and clinical data are summarised in Table 2. The SRD group comprised 27 participants, 23 women and 4 men, with a median age of 45.5 years (interquartile range: 37.4 to 54.8 years). One participant (4%) reported a history of tumours, and 12 participants (44%) reported chronic diseases, with hypertension being the most common (6 participants, 22%). The group included three smokers (11%) and 24 non-smokers (89%). Regarding physical activity, 13 participants (48%) reported none, while 14 participants engaged in various activities with varying durations and frequencies.

### 3.4. Comparison of Two Groups—Anthropometric and Biochemical Comparison

**Figure 2:**
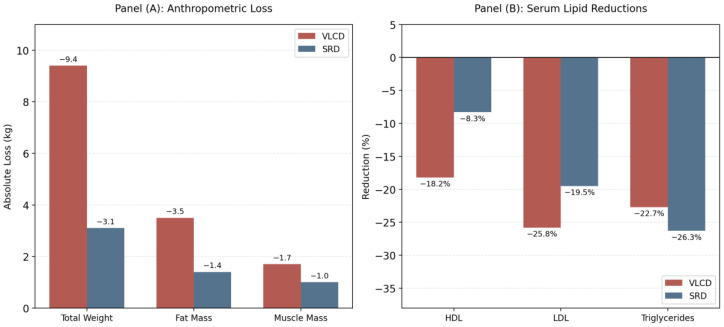
Non-parametric distribution matrices tracking changes in anthropometric and lipid biomarkers over the three-week study period. Panel (**A**) charts internal tissue compartment shifts, outlining median component mass loss (kg). Panel (**B**) highlights the median per cent reductions (%) calculated across main serum lipids. VLCD—Very Low-Calorie Diet group, SRD—Standard Reduced Diet group.

Table 3 and Figure 2 show significant baseline differences between the VLCD and SRD groups in mean age and BMI, with the VLCD group being older and having higher BMIs. Both groups experienced substantial within-group decreases in BMI following their respective diets (*p* < 0.001). The VLCD group demonstrated a significantly greater reduction in both weight (9.4 kg vs. 3.1 kg) and BMI (3.4 kg/m^2^ vs. 2.1 kg/m^2^) than the SRD group (median values). While the VLCD group had higher baseline values for weight, fat mass, and muscle mass, which remained elevated following the intervention, both diets resulted in a significant reduction in these parameters for the VLCD group: 3.5. kg (64.9 to median 61.4 kg, *p* < 0.001) and SRDs: 1.4 kg (58 to 56.6 kg, *p* < 0.001) for fat mass, as well as muscle mass for VLCDs: 1.7 kg (38.1 to 36.4 kg) and SRDs: 1 kg (32.5 to 31.5 kg), but not fat mass percentage: −1.1% for VLCD (46.4 to 47.5%) and 0.1% for SRD (50.3 to 50.2%). All those values were median differences.

When comparing medians, the VLCD group showed a significantly greater dynamic drop in body weight and BMI than the SRD group. While both groups experienced substantial within-group decreases in BMI following their respective diets (*p* < 0.001), the median reduction was more pronounced in the VLCD stream. Both diets resulted in a significant downward shift in total body fat mass and muscle mass compartments. The immediate post-intervention changes in overall fat percentage remained small over this short three-week window in both parallel cohorts.

Both groups exhibited a significant within-group reduction in fasting insulin levels following the three-week intervention (*p* < 0.001), for both cohorts. In parallel, from baseline (T0) to post-intervention (T1), median insulin values decreased by 4.4 mU/L in the VLCD group and by 2.2 mU/L in the SRD group.

Although baseline fasting glucose levels were higher in the VLCD group than in the SRD control group, the subsequent post-dietary decreases did not reach statistical significance within either individual cohort (*p* = 0.517) for VLCD and (*p* = 0.675) for SRD. Following the intervention phase, post-dietary median glucose values decreased by 2 mmol/L in the VLCD group and by 0.5 mmol/L in the SRD group.

Median leukocyte counts decreased significantly in both parallel arms (*p* < 0.001), decreasing by 0.4 × 10^9^/L in the VLCD and by 1 × 10^9^/L in the SRD group. In contrast, within-group variations for median high-sensitivity C-reactive protein (hsCRP) did not reach statistical significance in either intervention branch (*p* = 0.665) for VLCD; (*p* = 0.485) for SRD, even increasing by 0.4 mg/L in VLCD and decreasing by 1.3 mg/L in the SRD group.

The VLCD intervention resulted in a statistically significant decrease in all tracked lipid profile parameters (*p* < 0.001) (Table 3, Figure 2). Specifically, median LDL cholesterol dropped from 3.1 mmol/L to 2.3 mmol/L, median triglycerides decreased from 2.2 mmol/L to 1.7 mmol/L, and median HDL cholesterol shifted from 1.1 mmol/L to 0.9 mmol/L. Conversely, in the home-based SRD group, significant decreases occurred in only two lipid parameters: median LDL cholesterol dropped from 4.1 mmol/L to 3.3 mmol/L (*p* < 0.001) and median HDL cholesterol dropped from 1.2 mmol/L to 1.1 mmol/L (*p* < 0.001), while median triglycerides showed no statistically significant change, shifting from 1.9 mmol/L to 1.4 mmol/L (*p* = 0.571).

### 3.5. Food Questionnaire, DII and DNA Damage Biomarkers

FETA analysis of dietary questionnaires revealed that two participants in the experimental group had implausible energy intakes (479 kcal and 12,728 kcal) and were excluded [48]. Baseline dietary intake, assessed by the FETA program, showed no significant differences in energy, macronutrients, or micronutrients between the VLCD and SRD groups.

**Figure 3:**
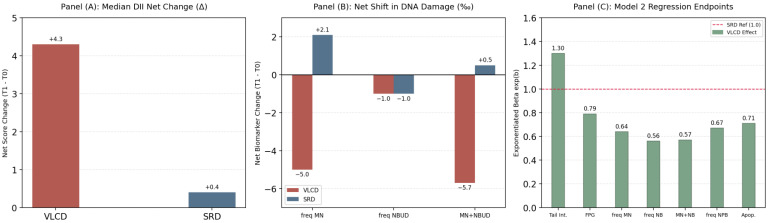
Longitudinal trajectories of dietary inflammatory potential, cytogenetic instability, and adjusted relative risk outcomes between VLCD and SRD cohorts over a three-week intervention. Panel (**A**) illustrates the median net change ∆ = T1 − T0 in the Dietary Inflammatory Index (DII) score. The pronounced positive shift observed in the very low-calorie diet (VLCD) cohort ∆ DII = +4.3) represents a mathematical artifact of non-selective food volume contraction inherent to the 600 kcal/day protocol, which, on paper, strictly reduces the absolute intake of anti-inflammatory micronutrients rather than signalling systemic inflammation in biological tissues. Panel (**B**) charts the net absolute shift in permanent chromosomal and structural genomic damage biomarkers, frequencies per 1000 binucleated cells, assessed via the cytokinesis-block micronucleus (CBMN) *cytome* assay. The hospital-supervised VLCD cohort exhibited highly significant within-group reductions across all indices, decreasing median micronuclei (freq MN) by −5.0‰, nuclear buds (freq NBUD) by −1.0‰, and the combined pooled biomarker index (MN + NBUD) by −5.7‰. In contrast, the home-based standard reduced diet (SRD) cohort remained statistically stagnant or shifted upward by +2.1‰ for freq MN and +0.5‰ for MN + NBUD, establishing the rapid genoprotective efficacy of an aggressive, medically monitored energy deficit. Panel (**C**) displays the exponentiated slope coefficients exp(b) derived from the fully adjusted multivariate regression models (Model 2). The horizontal reference line exp(b) = 1.0 isolates the baseline behaviour of the free-living SRD control group. Biomarkers with endpoints falling significantly below this unity line—specifically freq MN exp(b) = 0.643, 95%CI: 0.429–0.966 and freq NBUD exp(b) = 0.561, 95%CI: 0.160–1.954—demonstrate that the VLCD intervention exerts a powerful, independent protective effect against permanent genomic damage, fully preserving its statistical significance even after mathematically controlling for structural baseline disparities in age, gender, baseline damage loads, leukocytosis, and dynamic body composition changes ∆BMI. VLCD—Very Low-Calorie Diet group, SRD—Standard Reduced Diet group. NUBD is the same as NB. NPB—nucleoplasmic bridges, Tail Int-tail intensity in % from comet assay, FPG—tail intensity in % in Fpg modified comet assay.

Correspondingly, baseline DII values did not differ significantly between groups (Table 4, Figure 3). The DII for the VLCD group was calculated as the average daily food intake over the three weeks. In contrast, the data for the SRD group were derived from 24 h dietary recalls. The VLCD significantly increased DII compared with both its pre-intervention value and the SRD group’s, indicating a greater pro-inflammatory potential of the VLCD. The SRD intervention did not substantially change the DII. Figure 3 and Table 4 illustrate that the median Dietary Inflammatory Index (DII) score increased significantly in the VLCD group from a baseline median of 1.9 to a post-intervention median of 6.2 (*p* < 0.001), indicating a highly pro-inflammatory dietary potential on paper due to severe food volume restriction. Conversely, actual systemic biological inflammation decreased, as evidenced by a significant decline in median leukocyte counts (*p* < 0.001) and a significant reduction in parameters of permanent chromosomal DNA damage. The DII calculation heavily factors in the absolute intake volume of 45 distinct food parameters, including anti-inflammatory micronutrients (such as flavonoids, vitamins, and specific fatty acids). Because a 600 kcal/day VLCD regimen represents a massive, non-selective volume restriction, the absolute amounts of these protective micronutrients drop drastically on paper, mathematically driving the DII score upward into a “pro-inflammatory” designation. We will mention this again in the regression modelling part (Model 2).

Food Frequency Questionnaire (FFQ) data showed no statistically significant correlation between historical baseline dietary intake and DNA damage levels across either stream (Supplementary Tables S1–S6).

At baseline (T0), baseline chromosomal and structural DNA damage parameters were significantly more elevated in the parallel VLCD stream than in the SRD control stream across all three independent assays. Following the three-week intervention, within-group evaluations of the VLCD group revealed a highly significant downward shift in cytokinesis-block micronucleus (CBMN) *cytome* biomarkers (Table 4, Figure 3). Specifically, the median frequency of micronuclei plummets from 10‰ at baseline to 5‰ post-VLCD (*p* < 0.001), and median nuclear buds (NBUD) drop from 2.5‰ to 1.5‰ (*p* = 0.016) (Table 4, Figure 3). Conversely, the home-based SRD cohort did not exhibit a significant decrease in CBMN *cytome* indicators over this time window, with the median micronuclei frequency remaining statistically unchanged at 86.5‰ at baseline versus 8.6‰ post-intervention (*p* = 0.486). Given that nuclear buds represent biomarker precursor forms that can precede the complete formation of a micronucleus, we evaluated an aggregated pooled index category (MN + NBUD). This combined category confirmed that the VLCD intervention successfully reduced median baseline damage by nearly half, from 12.5‰ to 6.8‰ (*p* < 0.001), whereas baseline NBUD and MN frequency remained elevated in the SRD group before and after the diet. Cell proliferation analysis showed that the median nuclear division index (NDI) in the VLCD stream shifted slightly upward from 1.2 (IQR: 1.1–1.4) to 1.3 (IQR: 1.2–1.7) (*p* = 0.012), remaining below the standard reference baseline of ~2.0. In the SRD stream, the median NDI was 2.0 (IQR: 1.8–2.3) at baseline and 2.1 (IQR: 1.7–2.3) post-intervention (*p* = 0.584), both within normal reference boundaries throughout the study. Crucially, the VLCD intervention significantly reduced all baseline markers in the micronucleus assay, including the median frequency of apoptotic cells—which serves as an operational index for programmed cell death driven by an accumulation of unrepaired double-strand breaks—dropping from 8.5‰ to 4‰ (*p* < 0.001). The dynamic within-group variation in the SRD group shifted the median apoptotic frequency from 5‰ to 2‰ (*p* < 0.001).

Primary and oxidative DNA damage, measured via the alkaline comet assay, showed non-significant decreases in both groups, though VLCD post-intervention primary damage remained elevated above the 10–11% accepted healthy median range. While baseline oxidative damage was nearly double in the VLCD group, post-diet levels converged, with no standard, established control range for oxidative DNA lesions. Median tail intensity values for all metrics are detailed in Supplementary Table S7.

Spearman’s correlation coefficients (r) between the change in the DII (ΔDII = T1DII − T0DII) and the changes in DNA damage parameters (ΔT1 − T0) for both groups did not show statistically significant correlations with the DNA damage indicators, including MN, NBUDs, NPBs, apoptotic and necrotic cells, Tail Intensity Mean, and FPG.

### 3.6. Regression Analysis

Regression analyses were performed to investigate whether the observed differences between the two dietary groups remained significant after adjusting for potential confounders in a multivariate model. Seven models were developed to assess the effect of VLCD vs. SRD on various DNA damage parameters. The results of the basic and extended models (Model 1 and Model 2, respectively) are summarised in Table 5 and Figure 3.

To ensure our conclusions regarding DNA damage were not confounded by these differences, our multivariate regression models were designed to mathematically isolate the effect of the dietary interventions. In Model 1, we controlled for the baseline values of each dependent DNA biomarker. In Model 2 (extended adjustment), we fully adjusted for these differences by explicitly including changes in BMI (ΔBMI) and in metabolic parameters such as glucose. Because our sample size did not permit loading more than one covariate per 10 observations to avoid model overfitting, ΔBMI served as a robust, dynamic surrogate for the metabolic changes induced by the substantial energy deficit relative to their total daily energy expenditure (TDEE). The regression models confirmed that VLCD exerted a highly significant protective effect against permanent DNA damage (e.g., freqMN and freqNBUD), independent of these baseline variations.

Overall, the VLCD was associated with lower levels of DNA damage than the SRD, as shown in models adjusted for covariates identified in the univariate analyses.

Significant reductions in DNA damage were observed among participants following VLCD for the MN frequency (freqMN), NBUDs (freqNBUD), and the combined index (MN + NBUD).

The model for apoptotic cells showed no significant difference between diets in the basic model, but after adjustment for relevant covariates (Model 2), the VLCD showed a protective effect (mean ratio = exp(b) = 0.712).

The regression model with necrotic cells as the dependent variable could not be fitted because there were no events in the SRD group (see the following section). Because of this strong imbalance in necrotic cell frequency between groups and the predominance of zero values (>70%), a standard linear regression could not be performed. A binary logistic regression model was therefore tested (presence vs. absence of necrotic cells), but the model did not converge, preventing reliable estimation of the effects.

Also, Model 2 confirmed that the physical, systemic weight loss and metabolic restructuring induced by the VLCD group overrode any theoretical dietary inflammatory potential (DII), resulting in a highly statistically significant reduction in permanent chromosomal damage markers (freqMN and freqNBUD).

## 4. Discussion

### 4.1. Research Gap and Study Contribution

While the impact of overweight and obesity on DNA damage, as measured by both comet and micronucleus assays, is well documented [10,11,49,50,51,52], research on the effects of weight loss remains limited. This study uniquely investigates the effects of VLCD and SRD on DNA damage in individuals with BMI ≥ 35 kg/m^2^ after three weeks, addressing a significant gap in the literature. The paucity of research in this area may stem from the challenges of achieving sustained weight loss and detecting changes in DNA damage, which may require longer interventions or involve individuals with higher BMIs [53].

### 4.2. Comparative Effects of VLCD and SRD on Anthropometric and Biochemical Measurements and DNA Damage

To contextualise the genotoxic burden of our cohort, baseline values were benchmarked against historical reference ranges from large-scale public data and international validation projects (such as the hCOMET and International Micronucleus Project cohorts). A hCOMET cohort database of 19,320 subjects from 44 laboratories in 26 countries was analysed for the comet assay [54,55]. Healthy human populations typically exhibit a baseline Comet tail intensity below 10% (9% for alive healthy individuals, 12.4% for cancer-free individuals, 17.7% for cancer cases, and 18% for deceased individuals). From the HUMN database of 7000 subjects from 25 laboratories, baseline values for MN frequency range from 4 to 8 per 1000 binucleated cells (median 6.5, interquartile range 3–12 per 1000 binucleated cells) [56]. Our cohort’s baseline values—particularly the VLCD group’s tail intensity of 12.7% and MN frequency of 10‰—clearly mirror the baseline genomic instability characteristic of severe, comorbid chronic inflammation, providing a robust cross-sectional confirmation of obesity-induced DNA damage before dietary initialisation.

Our findings indicate that within-group evaluations of the VLCD group showed greater reductions in body weight, BMI, lipid profiles, and insulin resistance than in the SRD group. However, because of profound baseline inequalities, this study functions more as a parallel evaluation of two distinct, non-randomised clinical cohorts than as an unconfounded comparative trial, and claims of between-group superiority must be interpreted with caution. Notably, neither diet demonstrated superior efficacy in reducing primary (repairable) and oxidative DNA damage. The lack of impact on primary and oxidative DNA damage may be due to the linear relationship between BMI and DNA damage [10,11,12,51], compounded by the higher baseline oxidative stress in the VLCD group. Even after the diet, over 75% of the VLCD group still presented with Class III obesity, and their median BMI remained high at 46.1 kg/m^2^. This persistent adipose tissue volume continues to act as a significant source of systemic oxidative stress, which likely masks short-term reductions in oxidative DNA lesions and limits the dynamic upregulation of DNA repair pathways [56,57,58,59,60].

The distinct molecular handling of plant-derived versus animal-derived lipids provides an additional mechanistic layer explaining these shifts in genomic stability. Diets heavily dominated by animal fats (rich in saturated fatty acids like palmitic acid) can disrupt mitochondrial membrane fluidity, accelerate electron leakage, and spike intracellular reactive oxygen species (ROS) that directly induce single-strand DNA breaks [61]. Conversely, plant oils—particularly the monounsaturated fatty acids (MUFAs) and polyunsaturated fatty acids (PUFAs) utilised in our SRD design—serve as ligands for peroxisome proliferator-activated receptors (PPARs) [61,62]. This signalling pathway upregulates endogenous antioxidant defences, reducing the baseline systemic oxidative pressure on the genome [61]. By actively structuring our interventions to minimise saturated animal fats, we isolated the beneficial effects of metabolic weight loss from the potential confounding effects of lipid peroxidation-induced genotoxicity.

Furthermore, the baseline disparities in age and BMI between the groups (*p* = 0.001) inherently imply distinct initial metabolic baselines. The older VLCD cohort exhibited a higher baseline BMI, which translated into a higher absolute Basal Metabolic Rate (BMR), yet their sedentary inpatient setting minimised physical activity-related energy expenditure. Conversely, the younger SRD group maintained a regular, active lifestyle outside the hospital. In tandem with these physical differences, baseline comorbidity profiles and intervention settings differed substantially. The VLCD group presented with an advanced cluster of metabolic diseases, including type 2 diabetes (66%) and chronic hypertension, which are well-established drivers of baseline genomic instability. The choice of settings—strict inpatient hospitalisation for the VLCD cohort versus a free-living home environment for the SRD cohort—was a strict clinical and ethical mandate. A 600 kcal/day VLCD regimen requires 24/7 medical supervision to manage acute risks such as electrolyte shifts, whereas the standard deficit of the SRD cohort can be safely managed at home. While the inpatient setting effectively eliminated dietary non-compliance for the VLCD group, it introduced distinct environmental baselines and an immeasurable compliance bias. It remains impossible to fully isolate whether the superior outcomes observed in the VLCD group stem solely from the biological mechanism of a 600 kcal restriction or from the institutional environment that mathematically eliminated non-compliance. Our adjusted multivariate regression models (Model 2) confirmed that, despite these structural and setting disparities, the VLCD group’s profound reduction in permanent chromosomal damage markers (MN, NBUD, and NPB) remained independently significant. This demonstrates that severe caloric restriction fundamentally shifts genome stability in a highly specific, advanced clinical setting, extending beyond standard outpatient lifestyle weight-loss pathways alone.

### 4.3. Influence of VLCD on DNA Stability

A higher energy deficit, while leading to greater weight loss, can also increase the risk of nutrient deficiencies essential for DNA repair, de novo nucleotide synthesis, and antioxidant processes [14,15,16]. There is evidence that DNA repair, as measured by the comet assay, decreases with increasing BMI, particularly in individuals with BMI > 35 kg/m^2^. Base excision repair (BER) and nucleotide excision repair (NER), which are among the first DNA repair pathways to be suppressed with increases in BMI, can be reactivated with decreases in BMI and weight loss. Obesity-related factors increase DNA damage and impair DNA repair mechanisms. Adipokines, oxidative stress, inflammation, gut microbiota, and metabolic changes strongly influence DNA damage response (DDR) in obesity. Weight-loss interventions improve DDR function and reduce cancer risk [63]. It is known from human and in vivo studies, that obesity over 30 kg/m^2^ and especially over 35 starts to lower or decrease DNA repair mechanisms including base excision repair (BER), first mechanism that repairs DNA-single strand breaks, and nucleotide excision repair (NER)-bulky adducts, and there are three other DNA repair mechanisms-for base mismatch and two for severe double strand DNA breaks, but it is also known that decrease in BMI bellow 35 and better bellow 30 again activates those mechanisms back (bariatric surgery and in vivo) [64,65,66,67]. So, if the yo-yo effect were to occur and patients were to again reach BMI over 30 or 35, for sure, DNA repair mechanisms would show the same effects of decreasing/lowering or shutting down.

Furthermore, while higher energy deficits promote greater weight loss, they can also increase the risk of nutrient deficiencies that affect DNA repair [14,15,16] and can lead to a highly inflammatory state [68]. Although the VLCD contained ingredients with higher DII values, and the SRD did not alter DII values, only the SRD group exhibited elevated CRP levels after the diet. Nevertheless, we did not further explore this aspect in this study. Although our previous study demonstrated that the anti-inflammatory effect of food has a weak correlation with primary DNA damage, it is a strong predictor of DNA damage in individuals with obesity when considered alongside other parameters, such as lipid profile markers [69].

Our results also indicate that VLCD has a more favourable effect on permanent DNA damage than SRD. Consistent with this, the SRD group had higher baseline NBUD and MN + NBUD values than those on VLCD, potentially due to lower folate levels, which are essential for DNA repair and stability [17,70]. A deficiency in folic acid and/or vitamin B_12_ can cause the formation of MN and NBUD [17,65]. This theory is reinforced by the median folic acid concentration in the SRD group before the study being 11.7 nmol/L, which was significantly lower (*p* = 0.047) than in the VLCD group (16.65 nmol/L). The survey by Donmez-Altuntas et al. (2014) did not reveal differences in the frequency of MN, NPB, and NBUD among individuals with varying degrees of obesity [50]. However, participants with comorbidities, such as type 2 diabetes, were excluded from that study. The experimental group consisted of 66% participants who reported having diabetes.

The favourable impact of VLCD on glucose, insulin, and lipid status, as well as weight reduction [24,25], may contribute to decreased MN values, as high insulin levels have been linked to genomic damage [71,72,73], and diabetes/insulin resistance has been found to affect MN frequency [60]. In vitro studies have shown that elevated insulin levels can cause genomic damage and lead to MN formation [72,73]. Therefore, the substantial decrease in insulin and HOMA-IR could enhance DNA stability and reduce the frequency of MN. The lack of a favourable effect on MN values in the SRD group after the diet could also be explained by interindividual differences (polymorphisms) in DNA repair and folate metabolism, as well as by insulin levels [74].

An unmeasured confounding factor in our study is the psychological impact of aggressive dietary restriction. Hospitalisation combined with a literal drop to a 600 kcal/day regimen represents an acute lifestyle shock that can provoke psychological stress and anxiety. This psychological distress can activate the hypothalamic–pituitary–adrenal (HPA) axis, increasing systemic cortisol levels [75,76]. Because cortisol surges are fundamentally tied to elevated intracellular reactive oxygen species (ROS) and altered DNA repair enzyme kinetics [77], HPA axis activation could actively compete against the biological benefits of weight loss [78]. This may partially explain why primary and oxidative DNA damage, measured via the comet assay, did not see a statistically significant decrease during this highly dynamic 3-week phase, as the stress of the intervention may have temporarily masked the underlying reduction in oxidative genotoxicity. Cortisol can modulate the transcription of 21 genes directly related to DNA damage signalling pathways, including the up-regulation of DNA damage sensors Chk1 and Chk2 and the proto-oncogene CDC25A, which is involved in cell cycle delay following DNA damage [79,80]. Every intervention that can lower the frequency of permanent DNA damage, for example, in the shape of micronuclei, and increase cell cycle velocity, meaning that cells will normally repair damaged cells and not make the pool of non-stable tumorigenic ones, is a good intervention, although done in strict hospital conditions.

## 5. Conclusions and Future Perspectives

In conclusion, this non-randomised, quasi-experimental study demonstrates that acute, short-term dietary weight-loss interventions are associated with significant improvements in metabolic and genomic health indicators among individuals with severe Class III obesity. Within-group evaluations revealed that while a standard reduced-calorie diet (SRD) serves as an accessible, home-based tool for improving insulin sensitivity and lipid parameters, a short-term, hospital-supervised very low-calorie diet (VLCD) acts as a powerful molecular “reset” associated with a significant within-group reduction in markers of permanent chromosomal damage, effectively halving parameters such as micronuclei (freqMN). Clinically, these findings hold significant translational promise, demonstrating that acute energy deficits can rapidly halt severe genotoxic stress and mitigate chronic oxidative stress, even when overriding an advanced background of metabolic comorbidities and baseline age disparities.

However, due to major structural disparities in baseline age, chronic disease burdens, and institutional compliance environments between the cohorts, direct comparative superiority between the two dietary paradigms cannot be definitively established. These findings should be treated as exploratory observations, particularly because the biological benefits were observed during a highly dynamic weight-loss phase rather than at a stable body mass plateau.

Furthermore, VLCD interventions carry a well-documented clinical risk of post-discharge compensatory weight rebound (the “yo-yo effect”) when patients transition from strictly monitored inpatient settings to unmonitored environments. From a genomic perspective, it remains highly critical to determine whether the significant improvements observed in permanent genetic stability are sustained over the long term, or whether weight regain triggers a secondary wave of acute adipose tissue inflammation that renders these cellular benefits transients. Consequently, future randomised controlled trials with structured longitudinal tracking at 3, 6, and 12 months are mandatory to fully validate these parallel nutritional pathways and confirm whether short-term genomic stabilisation successfully translates into a permanent, long-term reduction in the risk of chronic metabolic diseases and malignancies. The study compares two distinct clinical pathways, and evaluating these parallel cohorts offers high ecological validity. It reflects how these interventions actually perform in clinical practice. The primary objective was to determine whether the rapid, aggressive metabolic shift induced by a VLCD yields a distinct genomic stability trajectory compared with a standard, gradual deficit (SRD)—despite baseline differences.

## Figures and Tables

**Table 1:** Demographic and health characteristics of the experimental (VLCD) group.

No	S	Age	Tumour	Chronic Diseases	Smoking	Physical Activity
1	F	60	No	No	No	No
2	M	55	No	HBP, dyslipidemia	No	No
3	F	64	Yes	DT2 HBP, dyslipidemia	No	No
4	M	48	No	Asthma, PTSP, dyslipidemia	No	Walking, cycling
5	M	66	No	DT2, HBP, dyslipidemia	No	Physical labor
6	F	65	No	Asthma, HBP, dyslipidemia	No	No
7	M	42	No	HBP, hypothyroidism	No	No
8	M	66	Yes	Hypothyroidism, HBP, dyslipidemia	No	No
9	M	51	No	No	No	No
10	F	61	Yes	DT2, HBP	No	No
11	F	52	No	HBP, DT2, dyslipidemia	Yes	Walking
12	M	55	No	Sinusitis, psoriasis, arthritis, DT2, HBP, dyslipidemia	No	Physical labour, cycling, dancing
13	F	56	Yes	Asthma, DT2, HBP, dyslipidemia	No	No
14	F	67	Yes	DT2, HBP, dyslipidemia	No	workout
15	M	57	No	DT2, HBP, dyslipidemia	No	No
16	F	46	No	DT2, HBP, dyslipidemia	No	No
17	M	50	No	DT2, HBP, dyslipidemia	No	Physical labour
18	M	68	No	DT2, HBP, dyslipidemia	No	Walking
19	M	59	No	DT2, HBP, osteoarthritis	No	No
20	F	61	Yes	DT2, HBP	No	No
21	M	41	No	DT2, HBP, dyslipidemia	Yes	No
22	F	64	Yes	DT2, HBP, dyslipidemia	No	Walking
23	F	64	Yes	DT2, HBP, dyslipidemia	No	Walking
24	M	43	No	DT2, HBP	No	No
25	F	58	No	DT2, HBP, atrial fibrillation, dyslipidemia, hyperuricemia	No	No
26	F	60	No	DT2, HBP, dyslipidemia, hypothyroidism	No	Walking

S—sex, F—female, M—male, HBP—High blood pressure (hypertension), DT2—Type 2 diabetes.

**Table 2:** Demographic and health characteristics of the SRD group.

No	S	Age	Tumour	Chronic Diseases	Smoking	Physical Activity
1	F	29	No	No	No	Yoga, walking
2	F	41	No	No	No	No
3	F	39	No	No	No	Walking
4	F	44	No	HBP	No	Walking
5	F	46	No	No	No	No
6	F	46	No	No	No	Walking
7	M	26	No	No	No	No
8	F	65	No	HBP	No	Walking
9	F	64	No	No	No	No
10	F	49	No	Endometriosis	No	Workout
11	F	36	No	No	No	Gym
12	F	41	No	No	No	Walking, hiking
13	M	51	No	No	No	Hiking
14	F	36	No	Hashimoto thyroiditis	Yes	Walking
15	F	63	No	No	No	Walking
16	F	35	No	No	No	Walking
17	F	30	No	Hypothyroidism	No	No
18	F	38	No	HBP, anxiety	Yes	No
19	F	61	No	No	No	No
20	F	48	Ovary	Hypothyroidism	No	No
21	F	58	No	Pulmonary Sarcoidosis, hypothyroidism, HBP	No	No
22	M	61	No	No	No	No
23	M	60	No	HBP	No	Walking, swimming
24	F	49	No	HBP	No	No
25	F	40	No	Asthma	No	No
26	F	46	No	anxiety, HBP, PCOS	Yes	No
27	F	27	No	No	No	Workout

S—sex, F—female, M—male, HBP—High blood pressure (hypertension), PCOS—Polycystic ovary syndrome.

**Table 3:** Descriptive and univariate analysis of anthropometric and biochemical parameters between the VLCD group vs. the standard 1500 kcal reduction diet (SRD).

Parameters	Before Intervention (T0)		*p* ^a^	After Intervention (T1)		*p* ^a^	VLCD T0-T1 *p* ^b^	SRD T0-T1 *p* ^b^
	VLCD Median (IQR)	SRD Median (IQR)		**VLCD** **Median (IQR)**	**SRD** **Median (IQR)**			
Age (yrs)	58.5 (13)	45.6 (22)	0.001	-	-	-	-	-
BMI (kg/m^2^)	49.4 (8.5)	42.1 (5.6)	0.001	46.0 (8.9)	40.0 (6.0)	0.009	<0.001	<0.001
Leukocytes (10^9^/L)	7.3 (2.8)	8.1 (2.3)	0.510	6.9 (2.2)	7.1 (2.0)	0.715	<0.001	<0.001
hsCRP (mg/L)	6.6 (12.7)	7.1 (6.8)	0.831	7.0 (10.2)	5.8 (8.6)	0.466	0.665	0.485
Glucose (mmol/L)	7.5 (3.1)	6.2 (1.4)	0.003	5.5 (2.1)	5.7 (1.4)	0.498	0.517	0.675
Insulin (mU/L)	16.4 (8.3)	14.0 (8.9)	0.831	12.0 (10)	11.8 (10)	0.943	<0.001	<0.001
HDL cholesterol (mmol/L)	1.1 (0.6)	1.2 (0.4)	0.561	0.9 (0.4)	1.1 (0.4)	0.075	<0.001	<0.001
LDL cholesterol (mmol/L)	3.1 (1.6)	4.1 (1.9)	0.111	2.3 (1.6)	3.3 (1.7)	0.001	<0.001	<0.001
Triglycerides (mmol/L)	2.2 (1.2)	1.9 (1.2)	0.121	1.7 (0.8)	1.4 (0.6)	0.256	<0.001	0.571

a: *p*-value Mann–Whitney; b: Wilcoxon signed rank; *p* < 0.05. Table updated from “mean ± SD” to “median (IQR—interquartile range)” for all biochemical, anthropometric, and genomic biomarkers to preserve absolute statistical consistency with the non-parametric tests employed. IQR is defined as the difference between the third quartile (Q_3_, the 75th percentile) and the first quartile (Q_1_, the 25th percentile). T0—baseline, T1—post-intervention after 3 weeks of diet. VLCD—Very Low-Calorie Diet group, SRD—Standard Reduced Diet group, BMI—body mass index, hsCRP— high-sensitivity C-reactive protein, HDL—high-density lipoprotein, LDL—low-density lipoprotein.

**Table 4:** Descriptive and univariate analysis of genome (DNA) stability between the VLCD group vs. SRD.

Parameters	Before Intervention (T0)		*p* ^a^	After Intervention (T1)		*p* ^a^	VLCD T0-T1 *p* ^b^	SRD T0-T1 *p* ^b^
	VLCD Median (IQR)	SRD Median (IQR)		VLCD Median (IQR)	SRD MEDIAN (IQR)			
DII	1.9 (3.2)	2.3 (2.5)	0.571	6.2 (0.0)	2.7 (3.5)	<0.001	<0.001	0.387
freq MN	10.0 (6.9)	6.5 (4.5)	0.008	5.0 (3.6)	8.6 (5.0)	0.005	<0.001	0.486
freq NBUD	2.5 (2.4)	8.0 (8.0)	<0.001	1.5 (2.0)	7.0 (7.0)	<0.001	0.016	0.106
MN + NBUD	12.5 (7.1)	14.0 (12.0)	0.702	6.8 (3.1)	14.5 (12.0)	<0.001	<0.001	0.435
freq NPB	5.0 (8.1)	4.5 (4.5)	0.277	2.3 (3.8)	3.0 (5.5)	0.292	0.002	0.375
apoptotic cells	8.5 (8.0)	5.0 (5.0)	0.026	4.0 (4.8)	2.0 (3.0)	0.129	<0.001	<0.001
necrotic cells	0.0 (8.0)	0.0 (0.0)	0.001	0.0 (5.0)	0 (0)	<0.001	0.683	0.317
Tail Intensity Mean	12.7 (5.3)	7.5 (4.8)	0.001	11.1 (6.0)	7.1 (5.1)	0.002	0.182	0.186
FPG mean of two medians	4.0 (8.7)	4.5 (6.5)	0.859	2.2 (7.7)	3.9 (4.5)	0.803	0.276	0.361

a: *p*-value Mann–Whitney; b: Wilcoxon signed rank; *p* < 0.05. Table updated from “mean ± SD” to “median (IQR—interquartile range)” for all biochemical, anthropometric, and genomic biomarkers to preserve absolute statistical consistency with the non-parametric tests employed. IQR is defined as the difference between the third quartile (Q_3_, the 75th percentile) and the first quartile (Q_1_, the 25th percentile), VLCD—Very Low-Calorie Diet group, SRD—Standard Reduced Diet group, DII—Dietary Inflammatory Index, MN—micronuclei, NBUD—nuclear bud, NPB—nucleoplasmic bridge, FPG—formamidopyrimidine DNA glycosylase.

**Table 5:** Results of the regression analysis showing observed differences between the two dietary groups after adjusting for potential confounders in multivariate models (Models 1 and 2).

Dependent Variable	Model Type	Adj	VLCD Versus SRD				r^2^
			b	(SE)	exp(b)	95%CI	
T1 Tail intensity mean	a	Model 1	0.454	(0.197)	1.575	(1.062; 2.337)	0.360
	a	Model 2 d	0.263	(0.252)	1.301	(0.783; 2.158)	0.474
T1 FPG mean of two medians	a	Model 1	−0.413	(0.363)	0.662	(0.318; 1.374)	0.329
	a	Model 2 e	−0.235	(0.029)	0.791	(0.365; 1.714)	0.380
T1freqMN	a	Model 1	−0.519	(0.177)	0.595	(0.417; 0.849)	0.384
	a	Model 2 f	−0.441	(0.202)	0.643	(0.429; 0.966)	0.476
T1freqNB	b	Model 1	−1.436	(0.501)	0.238	(0.087; 0.651)	0.403
	b	Model 2 g	−0.578	(0.619)	0.561	(0.160; 1.954)	0.627
T1MNNB	a	Model 1	−0.692	(0.178)	0.501	(0.350; 0.717)	0.481
	a	Model 2 h	−0.564	(0.288)	0.569	(0.319; 1.015)	0.496
T1freqNPB	b	Model 1	−0.274	(0.453)	0.760	(0.306; 1.887)	0.126
	b	Model 2 i	−0.408	(0.446)	0.665	(0.271; 1.632)	0.428
T1 apoptotic cells	b	Model 1	0.320	(0.551)	1.377	(0.455; 4.170)	0.330
	b	Model 2 j	−0.340	(0.550)	0.712	(0.235; 2.153)	0.543

a: Linear regression model with dependent variable log-transformed using the natural logarithm; b: Linear regression model with Logarithmic transformation applied after adding a constant of 0.01 to allow computation of ln(Y + 0.01) (Osborne, 2002 [45]); Model 1 (basic adjustment): adjustment based on VLCD group (vs. SRD = 0) and baseline value; Model2: Adjustment based on VLCD, baseline value and the first three most significant according to the univariate analyses: d: adjusted for VLCD, baseline value, ΔBMI (T1 − T0), glucose, zinc*; e: adjusted for VLCD, baseline value FPG, folic acid; f: adjusted for VLCD*, baseline value FreqMN, gender, leukocytes, glucose; g: adjusted for VLCD, baseline value FreqNBUD, ΔDII (T1 − T0), glucose*, vitamin B12*; h: adjusted for VLCD, baseline value MN + NBUD, gender, ΔDII (T1 − T0), glucose; i: adjusted for VLCD, baseline value NPB, HOMAIR, insulin, ALT; j: adjusted for VLCD, baseline value*, glucose*, creatinine*, vitamin B12*.

## Data Availability

The raw data supporting the conclusions of this article will be made available by the authors on request because of the ethical restrictions.

## Supplementary Materials

The following supporting information can be downloaded at: https://www.mdpi.com/article/10.3390/nu18121985/s1. Table S1: Spearman’s correlation between average intake of specific food groups (FFQ) and DNA damage parameters in the VLCD group, with statistical significance (*p* < 0.999); Table S2: Spearman’s correlation between average energy and nutrient intake (FFQ) and DNA damage parameters in the VLCD group, with statistical significance (*p* < 0.999); Table S3: Spearman’s correlation between average vitamin intake (FFQ) and DNA damage parameters in the VLCD group, with statistical significance (*p* < 0.999); Table S4: Spearman’s correlation between average intake of specific food groups (FFQ) and DNA damage parameters in the SRD group, with statistical significance (*p* < 0.999); Table S5: Spearman’s correlation between average energy and nutrient intake (FFQ) and DNA damage parameters in the SRD group, with statistical significance (*p* < 0.999); Table S6: Spearman’s correlation between average vitamin intake (FFQ) and DNA damage parameters in the SRD group, with statistical significance (*p* < 0.999); Table S7: Spearman’s correlation coefficient (r) between the change in Dietary Inflammatory Index (ΔDII) and the change in DNA damage parameters (Δ = T1 − T0) in the SRD and VLCD groups.

## Abbreviations

The following abbreviations are used in this manuscript:

BMI	body mass index
CBC	complete blood count
DII	Dietary Inflammatory Index
FFQ	food frequency questionnaire
FPG	formamidopyrimidine DNA glycosylase
HOMA-IR	Homeostatic Model Assessment for Insulin Resistance
hsCRP	high-sensitivity C-reactive protein
MN	micronuclei
NBUD	nuclear bud
NPB	nucleoplasmic bridges
ROS	reactive oxygen species
SRD	Standard Reducing Diet
VLCD	very low-calorie diet
WHO	World Health Organisation
DDR	DNA damage response
BER	base excision repair
NER	Nucleotide excision repair
TI	Tail Intensity, % DNA in comet tail
TDEE	total daily energy expenditure

## References

[1] World Health Organisation (2025). International Classification of Diseases for Mortality and Morbidity Statistics.

[2] Sharma A.M., Kushner R.F. (2009). A proposed clinical staging system for obesity. Int. J. Obes..

[3] Keys A., Fidanza F., Karvonen M.J., Kimura N., Taylor H.L. (1972). Indices of relative weight and obesity. J. Chronic Dis..

[4] World Health Organisation (2024). One in Eight People are Now Living with Obesity. https://www.who.int/news/item/01-03-2024-one-in-eight-people-are-now-living-with-obesity.

[5] Eurostat (2024). Overweight and Obesity—BMI Statistics. https://ec.europa.eu/eurostat/statistics-explained/index.php?title=Overweight_and_obesity_-_BMI_statistics.

[6] Tangvarasittichai S. (2015). Oxidative stress, insulin resistance, dyslipidemia and type 2 diabetes mellitus. World J. Diabetes.

[7] Usman M., Volpi E.V. (2018). DNA damage in obesity: Initiator, promoter and predictor of cancer. Mutat. Res./Rev. Mutat. Res..

[8] Włodarczyk M., Nowicka G. (2019). Obesity, DNA Damage, and Development of Obesity-Related Diseases. Int. J. Mol. Sci..

[9] Nilsson R., Liu N.A. (2020). Nuclear DNA damages generated by reactive oxygen molecules (ROS) under oxidative stress and their relevance to human cancers, including ionizing radiation-induced neoplasia part I: Physical, chemical and molecular biology aspects. Radiat. Med. Prot..

[10] Bukhari S.A., Rajoka M.I., Nagra S.A., Rehman Z.U. (2010). Plasma homocysteine and DNA damage profiles in normal and obese subjects in the Pakistani population. Mol. Biol. Rep..

[11] Luperini B.C.O., Almeida D.C., Porto M.P., Marcondes J.P.C., Prado R.P., Rasera I., Oliveira M.R.M., Salvadori D.M.F. (2015). Gene polymorphisms and increased DNA damage in morbidly obese women. Mutat. Res./Fundam. Mol. Mech. Mutagen..

[12] Bankoglu E.E., Mukama T., Katzke V., Stipp F., Johnson T., Kühn T., Seyfried F., Godschalk R., Collins A., Kaaks R. (2022). Short- and long-term reproducibility of the COMET assay for measuring DNA damage biomarkers in frozen blood samples of the EPIC-Heidelberg cohort. Mutat. Res./Genet. Toxicol. Environ. Mutagen..

[13] Crespo-Orta I., Ortiz C., Encarnación J., Suárez E., Matta J. (2023). Association between DNA repair capacity and body mass index in women. Mutat. Res./Mol. Mech. Mutagen..

[14] Kaźmierczak-Barańska J., Boguszewska K., Karwowski B.T. (2020). Nutrition Can Help DNA Repair in the Case of Aging. Nutrients.

[15] Del Bo’ C., Martini D., Bernardi S., Gigliotti L., Marino M., Gargari G., Meroño T., Hidalgo-Liberona N., Andres-Lacueva C., Kroon P.A. (2021). Association between Food Intake, Clinical and Metabolic Markers and DNA Damage in Older Subjects. Antioxidants.

[16] Fenech M. (2020). The role of nutrition in DNA replication, DNA damage prevention and repair. Principles of Nutrigenetics and Nutrigenomics.

[17] Ladeira C., Gomes M.C., Brito M. (2014). Human nutrition, DNA damage and cancer: A review. Mutagenesis: Exploring Novel Genes and Pathways.

[18] Gaskin C.J., Cooper K., Stephens L.D., Peeters A., Salmon J., Porter J. (2024). Clinical practice guidelines for the management of overweight and obesity published internationally: A scoping review. Obes. Rev..

[19] Durrer Schutz D., Busetto L., Dicker D., Farpour-Lambert N., Pryke R., Toplak H., Widmer D., Yumuk V., Schutz Y. (2019). European Practical and Patient-Centred Guidelines for Adult Obesity Management in Primary Care. Obes. Facts.

[20] Yumuk V., Tsigos C., Fried M., Schindler K., Busetto L., Micic D., Toplak H. (2015). European guidelines for obesity management in adults. Obes. Facts.

[21] (2025). Obesity: Identification, Assessment and Management.

[22] Garvey W.T., Mechanick J.I., Brett E.M., Garber A.J., Hurley D.L., Jastreboff A.M., Nadolsky K., Pessah-Pollack R., Plodkowski R. (2016). American association of clinical endocrinologists and American college of endocrinology comprehensive clinical practice guidelines for medical care of patients with obesity. Endocr. Pract..

[23] Sumithran P., Proietto J., Gill T. (2015). Very-low-calorie diets (VLCDs) for the treatment of obesity. Managing and Preventing Obesity.

[24] Franz M.J., VanWormer J.J., Crain A.L., Boucher J.L., Histon T., Caplan W., Bowman J.D., Pronk N.P. (2007). Weight-loss outcomes: Systematic review and meta-analysis of weight-loss clinical trials with a Minimum 1-Year Follow-Up. J. Am. Diet. Assoc..

[25] Škrha J., Kunešová M., Hilgertová J., Weiserová H., Křížová J., Kotrlíková E. (2005). Short-term very-low-calorie diet reduces oxidative stress in obese type 2 diabetics. Physiol. Res..

[26] Mraz M., Lacinova Z., Drapalova J., Haluzikova D., Horinek A., Matoulek M., Trachta P., Kavalkova P., Svacina S., Haluzik M. (2011). The Effect of Very-Low-Calorie Diet on mRNA Expression of Inflammation-Related Genes in Subcutaneous Adipose Tissue and Peripheral Monocytes of Obese Patients with Type 2 Diabetes Mellitus. J. Clin. Endocrinol. Metab..

[27] Román-Pintos L.M., Villegas-Rivera G., Cardona-Muñoz E.G., Rodríguez-Carrizalez A.D., Moreno-Ulloa A., Rubin N., Miranda-Díaz A.G. (2018). Very Low-Calorie Diets in Type 2 Diabetes Mellitus: Effects on Inflammation, Clinical and Metabolic Parameters. Diabetes and Its Complications.

[28] Del Corral P., Chandler-Laney P.C., Casazza K., Gower B.A., Hunter G.R. (2009). Effect of dietary adherence with or without exercise on weight loss: A mechanistic approach to a global problem. J. Clin. Endocrinol. Metab..

[29] Ožvald I., Božičević D., Duh L., Vinković Vrček I., Domijan A.M., Milić M. (2022). Changes in anthropometric, biochemical, oxidative, and DNA damage parameters after 3-weeks-567-kcal-hospital-controlled-VLCD in severely obese patients with BMI ≥ 35 kg m^−2^. Clin. Nutr. ESPEN.

[30] Ožvald I., Božičević D., Duh L., Vinković Vrček I., Pavičić I., Domijan A.M., Milić M. (2021). Effects of a 3-Week Hospital-Controlled Very-Low-Calorie Diet in Severely Obese Patients. Nutrients.

[31] Soares N.P., Santos A.C.S., Costa E.C., Azevedo G.D., Damasceno D.C., Fayh A.P.T., Lemos T.M.A.M. (2016). Diet-Induced Weight Loss Reduces DNA Damage and Cardiometabolic Risk Factors in Overweight/Obese Women with Polycystic Ovary Syndrome. Ann. Nutr. Metab..

[32] Simundic A.M., Bölenius K., Cadamuro J., Church S., Cornes M.P., van Dongen-Lases E.C., Eker P., Erdeljanovic T., Grankvist K., Guimaraes J.T. (2018). EFLM-COLABIOCLI recommendation for venous blood sampling. Clin. Chem. Lab. Med..

[33] Friedewald W.T., Levy R.I., Fredrickson D.S. (1972). Estimation of the concentration of low-density lipoprotein cholesterol in plasma, without use of the preparative ultracentrifuge. Clin. Chem..

[34] Milić M., Ožvald I., Vinković Vrček I., Vučić Lovrenčić M., Oreščanin V., Bonassi S., Del Castillo E.R. (2019). Alkaline comet assay results on fresh and one-year frozen whole blood in small volume without cryo-protection in a group of people with different health status. Mutat. Res./Genet. Toxicol. Environ. Mutagen..

[35] Collins A., Møller P., Gajski G., Vodenková S., Abdulwahed A., Anderson D., Bankoglu E.E., Bonassi S., Boutet-Robinet E., Brunborg G. (2023). Measuring DNA modifications with the comet assay: A compendium of protocols. Nat. Protoc..

[36] Eastmond D.A., Tucker J.D. (1989). Identification of aneuploidy-inducing agents using cytokinesis-blocked human lymphocytes and an antikinetochore antibody. Environ. Mol. Mutagen..

[37] Fenech M. (2000). The in vitro micronucleus technique. Mutat. Res..

[38] Boone C., Judge S., Shami A., Danna B., Ball A.B., Waingankar T.P., Saqub H., Divakaruni A.S., Lewis S.C. (2025). Saturated lipid stress attenuates mitochondrial genome synthesis in human cells. bioRxiv.

[39] Mulligan A.A., Luben R.N., Bhaniani A., Parry-Smith D.J., O’Connor L., Khawaja A.P., Forouhi N.G., Khaw K.T. (2014). EPIC-Norfolk FFQ Study. A new tool for converting food frequency questionnaire data into nutrient and food group values: FETA research methods and availability. BMJ Open.

[40] Shivappa N., Steck S.E., Hurley T.G., Hussey J.R., Hébert J.R. (2014). Designing and developing a literature-derived, population-based dietary inflammatory index. Public Health Nutr..

[41] Neveu V., Perez-Jiménez J., Vos F., Crespy V., du Chaffaut L., Mennen L., Knox C., Eisner R., Cruz J., Wishart D. (2010). Phenol-Explorer: An online comprehensive database on polyphenol contents in foods. Database.

[42] Haytowitz D., Wu X., Bhagwat S. (2018). USDA Database for the Flavonoid Content of Selected Foods, Release 3.3. USDA Agricultural Research Service [Internet]. https://www.ars.usda.gov/northeast-area/beltsville-md-bhnrc/beltsville-human-nutrition-research-center/methods-and-application-of-food-composition-laboratory/mafcl-site-pages/flavonoids/.

[43] DTU (2015). Frida Food Data.

[44] Rothwell J.A., Perez-Jimenez J., Neveu V., Medina-Remón A., M’hiri N., García-Lobato P., Manach C., Knox C., Eisner R., Wishart D.S. (2013). Phenol-Explorer 3.0: A major update of the Phenol-Explorer database to incorporate data on the effects of food processing on polyphenol content. Database.

[45] Osborne J. (2002). Notes on the use of data transformations. Pract. Assess. Res. Eval..

[46] Mickey R.M., Greenland S. (1989). Impact of confounder selection criteria. Am. J. Epidemiol..

[47] Peduzzi P., Concato J., Kemper E., Holford T.R., Feinstein A.R. (1996). A simulation study of the number of events per variable in logistic regression analysis. J. Clin. Epidemiol..

[48] Willett W. (2013). Nutritional Epidemiology.

[49] Hofer T., Karlsson H.L., Möller L. (2006). DNA oxidative damage and lifestyle factors. Free Radic. Res..

[50] Donmez-Altuntas H., Sahin F., Bayram F., Bitgen N., Mert M., Guclu K., Hamurcu Z., Arıbas S., Gundogan K., Diri H. (2014). Evaluation of chromosomal damage, cytostasis, cytotoxicity, oxidative DNA damage and their association with body-mass index in obese subjects. Mutat. Res./Genet. Toxicol. Environ. Mutagen..

[51] Włodarczyk M., Jabłonowska-Lietz B., Olejarz W., Nowicka G. (2018). Anthropometric and Dietary Factors as Predictors of DNA Damage in Obese Women. Nutrients.

[52] Santovito A., Gendusa C. (2020). Micronuclei frequency in peripheral blood lymphocytes of healthy subjects living in Turin (North-Italy): Contribution of body mass index, age and sex. Ann. Hum. Biol..

[53] Bankoglu E.E., Seyfried F., Arnold C., Soliman A., Jurowich C., Germer C.T., Otto C., Stopper H. (2018). Reduction of DNA damage in peripheral lymphocytes of obese patients after bariatric surgery-mediated weight loss. Mutagenesis.

[54] Milić M., Ceppi M., Bruzzone M., Azqueta A., Brunborg G., Godschalk R., Koppen G., Langie S., Møller P., Teixeira J.P. (2021). The hCOMET project: International database comparison of results with the comet assay in human biomonitoring. Baseline frequency of DNA damage and effect of main confounders. Mutat. Res./Rev. Mutat. Res..

[55] Bonassi S., Ceppi M., Møller P., Azqueta A., Milić M., Neri M., Brunborg G., Godschalk R., Koppen G., Langie S.A.S. (2021). DNA damage in circulating leukocytes measured with the comet assay may predict the risk of death. Sci. Rep..

[56] Bonassi S., Fenech M., Lando C., Lin Y.P., Ceppi M., Chang W.P., Holland N., Kirsch-Volders M., Zeiger E., Ban S. (2001). HUman MicroNucleus project: International database comparison for results with the cytokinesis-block micronucleus assay in human lymphocytes: I. Effect of laboratory protocol, scoring criteria, and host factors on the frequency of micronuclei. Environ. Mol. Mutagen..

[57] Serafim M.P., Santo M.A., Gadducci A.V., Scabim V.M., Cecconello I., de Cleva R. (2019). Very low-calorie diet in candidates for bariatric surgery: Change in body composition during rapid weight loss. Clinics.

[58] Faria S.L., Faria O., Cardeal M.D.A., Ito M.K. (2015). Effects of a very low calorie diet in the preoperative stage of bariatric surgery: A randomized trial. Surg. Obes. Relat. Dis..

[59] Merino J., Megias-Rangil I., Ferré R., Plana N., Girona J., Rabasa A., Aragonés G., Cabré A., Bonada A., Heras M. (2013). Body weight loss by very-low-calorie diet program improves small artery reactive hyperemia in severely obese patients. Obes. Surg..

[60] Bankoglu E.E., Arnold C., Hering I., Hankir M., Seyfried F., Stopper H. (2018). Decreased Chromosomal Damage in Lymphocytes of Obese Patients After Bariatric Surgery. Sci. Rep..

[61] Sullivan E.M., Pennington E.R., Green W.D., Beck M.A., Brown D.A., Shaikh S.R. (2018). Mechanisms by Which Dietary Fatty Acids Regulate Mitochondrial Structure-Function in Health and Disease. Adv. Nutr..

[62] Bordoni A., Di Nunzio M., Danesi F., Biagi P.L. (2006). Polyunsaturated fatty acids: From diet to binding to PPARs and other nuclear receptors. Genes Nutr..

[63] Deshmukh B., Ajay A.K., Bhat M.K. (2026). Obesity and cancer: Relevance of DNA damage response. Transl. Oncol..

[64] Ferk F., Mišík M., Ernst B., Prager G., Bichler C., Mejri D., Gerner C., Bileck A., Kundi M., Langie S. (2023). Impact of Bariatric Surgery on the Stability of the Genetic Material, Oxidation, and Repair of DNA and Telomere Lengths. Antioxidants.

[65] Zheng C., Shaposhnikov S., Collins A., Brunborg G., Azqueta A., Langie S.A.S., Dusinska M., Slyskova J., Vodicka P., van Schooten F.J. (2025). A pooled analysis of host factors that affect nucleotide excision repair in humans. Mutagenesis.

[66] Opattova A., Langie S.A.S., Milic M., Collins A., Brevik A., Coskun E., Dusinska M., Gaivão I., Kadioglu E., Laffon B. (2022). A pooled analysis of molecular epidemiological studies on modulation of DNA repair by host factors. Mutat. Res./Genet. Toxicol. Environ. Mutagen..

[67] Setayesh T., Mišík M., Langie S.A.S., Godschalk R., Waldherr M., Bauer T., Leitner S., Bichler C., Prager G., Krupitza G. (2019). Impact of Weight Loss Strategies on Obesity-Induced DNA Damage. Mol. Nutr. Food Res..

[68] Liang S., Nasir R.F., Bell-Anderson K.S., Toniutti C.A., O’Leary F.M., Skilton M.R. (2022). Biomarkers of dietary patterns: A systematic review of randomized controlled trials. Nutr. Rev..

[69] Milić M., Ožvald I., Matković K., Radašević H., Nikolić M., Božičević D., Duh L., Matovinović M., Bituh M. (2023). Combined Approach: FFQ, DII, Anthropometric, Biochemical and DNA Damage Parameters in Obese with BMI ≥ 35 kg m^−2^. Nutrients.

[70] Fenech M. (2020). Cytokinesis-Block Micronucleus Cytome Assay Evolution into a More Comprehensive Method to Measure Chromosomal Instability. Genes.

[71] Othman E.M., Hintzsche H., Stopper H. (2014). Signaling steps in the induction of genomic damage by insulin in colon and kidney cells. Free Radic. Biol. Med..

[72] Othman E.M., Leyh A., Stopper H. (2013). Insulin mediated DNA damage in mammalian colon cells and human lymphocytes in vitro. Mutat. Res..

[73] Othman E.M., Kreissl M.C., Kaiser F.R., Arias-Loza P.A., Stopper H. (2013). Insulin-mediated oxidative stress and DNA damage in LLC-PK1 pig kidney cell line, female rat primary kidney cells, and male ZDF rat kidneys in vivo. Endocrinology.

[74] Wagner K.H., Schwingshackl L., Draxler A., Franzke B. (2021). Impact of dietary and lifestyle interventions in elderly or people diagnosed with diabetes, metabolic disorders, cardiovascular disease, cancer and micronutrient deficiency on micronuclei frequency—A systematic review and meta-analysis. Mutat. Res./Rev. Mutat. Res..

[75] Lengton R., Schoenmakers M., Penninx B.W.J.H., Boon M.R., van Rossum E.F.C. (2025). Glucocorticoids and HPA axis regulation in the stress-obesity connection: A comprehensive overview of biological, physiological and behavioural dimensions. Clin. Obes..

[76] Bose M., Oliván B., Laferrère B. (2009). Stress and obesity: The role of the hypothalamic-pituitary-adrenal axis in metabolic disease. Curr. Opin. Endocrinol. Diabetes Obes..

[77] Flaherty R.L., Owen M., Fagan-Murphy A., Intabli H., Healy D., Patel A., Allen M.C., Patel B.A., Flint M.S. (2017). Glucocorticoids induce production of reactive oxygen species/reactive nitrogen species and DNA damage through an iNOS mediated pathway in breast cancer. Breast Cancer Res..

[78] Appelhans B.M., Pagoto S.L., Peters E.N., Spring B.J. (2010). HPA axis response to stress predicts short-term snack intake in obese women. Appetite.

[79] Black C.N., Bot M., Révész D., Scheffer P.G., Penninx B. (2017). The association between three major physiological stress systems and oxidative DNA and lipid damage. Psychoneuroendocrinology.

[80] Flint M.S., Baum A., Chambers W.H., Jenkins F.J. (2007). Induction of DNA damage.; alteration of DNA repair and transcriptional activation by stress hormones. Psychoneuroendocrinology.

