# Supplementary material for: Effects of Caloric Restriction on DNA Damage: A Comparison of Very Low-Calorie and Standard Reduced-Calorie Diets in Obesity—Non-Randomised, Quasi-Experimental Clinical Intervention Study
Source: Nutrients. 2026 Jun 19;18(12):1985. doi: 10.3390/nu18121985 (PMC13306159; doi:10.3390/nu18121985)
Supplement: Supplementary file 1 [file nutrients-18-01985-s001.zip › nutrients-4357078-supplementary.pdf]

Supplementary Tables

Supplementary Table S1. Spearman's correlation between average intake of specific food groups (FFQ) and DNA damage parameters in the VLCD group with statistical significance ($p < 0.999$)

	FPG	Comet	MN	NBUD	NPB	NDI	Apoptotic cells
Alcoholic beverages (g)	0.02	0.00	0.16	0.10	−0.04	0.29	−0.32
Cereals & cereal products (g)	−0.32	−0.21	−0.13	−0.10	0.03	−0.07	−0.30
Eggs & egg dishes (g)	−0.04	0.19	0.23	0.33	0.10	0.15	0.00
Fats & oils (g)	−0.16	0.12	−0.20	0.10	0.14	0.20	0.43
Fish & fish products (g)	−0.13	−0.08	0.17	0.32	0.33	0.15	−0.33
Fruit (g)	0.05	0.13	0.16	−0.18	0.07	−0.49	−0.04
Meat & meat products (g)	0.02	−0.21	−0.01	−0.17	0.06	−0.23	−0.37
Milk & milk products (g)	−0.28	−0.02	−0.13	−0.46	0.03	0.03	−0.03

	FPG	Comet	MN	NBUD	NPB	NDI	Apoptotic cells
Non-alcoholic beverages (g)	−0.18	−0.14	0.08	−0.40	0.08	0.07	−0.24
Nuts & seeds (g)	−0.03	0.24	−0.44	0.07	−0.21	0.20	0.18
Potatoes (g)	0.00	0.21	−0.26	−0.01	−0.13	0.05	−0.20
Soups & sauces (g)	−0.28	−0.10	−0.13	0.19	0.37	−0.02	−0.02
Sugars, preserves & snacks (g)	−0.04	−0.33	−0.03	0.29	0.42	−0.39	−0.08
Vegetables (g)	−0.14	0.10	−0.25	0.08	0.19	−0.38	−0.17

FPG – formamidopyrimidine-DNA glycosylase; MN – micronuclei; NBUD – nuclear bud; NPB – nucleoplasmic bridge; NDI − nuclear division indeks; FFQ − Food Frequency Questionnaire

Supplementary Table S2. Spearman's correlation between average energy and nutrient intake (FFQ) and DNA damage parameters in the VLCD group with statistical significance ($p < 0.999$)

	FPG	Comet	MN	NBUD	NPB	NDI	Apoptotic cells
Energy (kcal)	−0.21	−0.23	−0.01	−0.06	0.34	−0.25	−0.53
Carbohydrate – total (g)	−0.35	−0.21	−0.04	−0.05	0.21	−0.33	−0.43
Carbohydrate - sugars (g)	−0.24	−0.07	0.11	−0.17	0.17	−0.35	−0.27
Protein (g)	−0.25	−0.06	−0.03	−0.05	0.30	−0.36	−0.55
Fat – total (g)	−0.07	−0.09	−0.12	0.13	0.40	−0.06	−0.36
MUFA (g)	−0.01	0.01	−0.11	0.15	0.31	0.00	−0.36
PUFA (g)	0.03	−0.01	−0.09	0.23	0.37	0.06	−0.31
SFA (g)	−0.09	−0.12	−0.13	0.08	0.40	−0.07	−0.30
Cholesterol (mg)	−0.15	−0.02	0.06	0.19	0.48	−0.10	−0.31
Alcohol (g)	0.07	0.00	0.25	0.11	−0.01	0.39	−0.27
Sodium (mg)	−0.14	−0.15	−0.23	0.10	0.22	0.13	−0.23
Chloride (mg)	−0.16	−0.12	−0.28	0.06	0.20	0.15	−0.22
Calcium (mg)	−0.32	−0.06	−0.13	−0.14	0.23	−0.18	−0.45
Phosphorus (mg)	−0.26	−0.09	−0.10	−0.12	0.35	−0.26	−0.44

	FPG	Comet	MN	NBUD	NPB	NDI	Apoptotic cells
Magnesium (mg)	−0.17	0.01	−0.10	−0.10	0.40	−0.21	−0.44
Potassium (mg)	−0.08	0.04	−0.12	−0.14	0.19	−0.28	−0.45
Iron (mg)	−0.06	−0.03	−0.11	0.00	0.42	−0.08	−0.34
Copper (mg)	0.10	−0.22	0.19	0.35	0.56	0.24	−0.24
Zinc (mg)	−0.23	−0.06	−0.10	−0.24	0.22	−0.20	−0.38
Selenium (μg)	−0.23	−0.21	0.05	0.07	0.34	−0.10	−0.38
Manganese(mg)	−0.31	0.07	−0.46	−0.08	0.21	0.03	−0.03

FPG – formamidopyrimidine-DNA glycosylase; MN – micronuclei; NBUD – nuclear bud; NPB – nucleoplasmic bridge; NDI − nuclear division indeks; MUFA i PUFA − monounsaturated fatty acids and. polyunsaturated fatty acids; SFA - Saturated Fatty Acids; FFQ − Food Frequency Questionnaire

Supplementary Table S3. Spearman's correlation between average vitamin intake (FFQ) and DNA damage parameters in the VLCD group with statistical significance ($p < 0.999$)

	FPG	Comet	MN	NBUD	NPB	NDI	Apoptotic cells
α carotene (µg)	−0.19	−0.05	−0.29	0.31	0.22	−0.37	−0.23
β carotene (µg)	−0.25	0.21	−0.20	−0.12	0.05	−0.40	−0.23
Vitamin A – retinol (µg)	0.04	−0.07	0.25	0.29	0.48	0.32	0.02
Vitamin B2 – riboflavin (mg)	−0.11	−0.12	0.06	0.05	0.42	0.05	−0.20
Vitamin B1 – thiamin (mg)	0.01	−0.02	−0.16	0.12	0.04	−0.03	−0.25
Vitamin B12 – cobalamin (µg)	−0.02	−0.01	0.21	0.06	0.46	0.28	−0.14
Total folate (µg)	−0.25	0.03	−0.22	0.03	0.27	−0.22	−0.29
Niacin (mg)	−0.14	0.00	−0.06	−0.02	0.26	−0.11	−0.41
Vitamin B6 – pyridoxine (mg)	−0.11	0.10	−0.17	−0.12	0.13	−0.40	−0.44
Vitamin C - ascorbic acid (mg)	−0.24	0.10	0.02	−0.30	0.01	−0.36	−0.28

	FPG	Comet	MN	NBUD	NPB	NDI	Apoptotic cells
Vitamin D – ergocalciferol (µg)	−0.04	0.01	0.00	−0.07	0.23	0.10	−0.30
Vitamin E - alpha tocopherol (mg)	−0.02	−0.03	−0.08	0.29	0.40	−0.12	−0.32

FPG – formamidopyrimidine-DNA glycosylase; MN – micronuclei; NBUD – nuclear bud; NPB – nucleoplasmic bridge; NDI − nuclear division indeks; FFQ − Food Frequency Questionnaire

Supplementary Table S4. Spearman's correlation between average intake of specific food groups (FFQ) and DNA damage parameters in the SRD group with statistical significance ($p < 0.999$)

	FPG	Comet	MN	NBUD	NPB	NDI	Apoptotic cells
Alcoholic beverages (g)	0.40	−0.01	−0.13	−0.07	0.31	0.01	−0.03
Cereals & cereal products (g)	−0.39	−0.10	0.01	0.02	−0.15	0.12	−0.14
Eggs & egg dishes (g)	−0.09	−0.02	0.31	0.27	0.07	−0.07	−0.22
Fats and oils (g)	−0.09	−0.12	−0.04	0.10	−0.05	0.20	−0.07
Fish & fish products (g)	−0.55	0.12	0.18	0.27	0.10	−0.50	0.08
Fruit (g)	−0.17	0.01	0.26	0.22	0.28	−0.25	0.13
Meat & meat products (g)	0.11	0.12	−0.15	−0.04	0.01	−0.08	−0.12
Milk & milk products (g)	−0.06	0.42	0.13	0.19	0.08	−0.20	0.09
Non-alcoholic beverages (g)	−0.17	0.05	−0.01	0.24	0.13	−0.47	0.15
Nuts & seeds (g)	−0.17	0.17	0.10	0.20	−0.09	−0.07	0.18
Potatoes (g)	−0.01	−0.21	−0.31	−0.29	0.36	0.07	−0.22

	FPG	Comet	MN	NBUD	NPB	NDI	Apoptotic cells
Soups & sauces (g)	−0.24	−0.02	0.00	0.49	0.13	−0.11	0.16
Sugars, preserves & snacks (g)	−0.18	−0.14	−0.16	−0.28	0.05	0.10	0.02
Vegetables (g)	−0.23	0.04	0.17	0.09	0.11	0.05	−0.13

FPG – formamidopyrimidine-DNA glycosylase; MN – micronuclei; NBUD – nuclear bud; NPB – nucleoplasmic bridge; NDI − nuclear division indeks; FFQ − Food Frequency Questionnaire

Supplementary Table S5. Spearman’s correlation between average energy and nutrient intake (FFQ) and DNA damage parameters in the SRD group with statistical significance ($p < 0.999$)

	FPG	Comet	MN	NBUD	NPB	NDI	Apoptotic cells
Energy (kcal)	−0.23	−0.10	0.06	0.08	0.15	0.13	0.03
Carbohydrate – total (g)	−0.26	−0.18	0.02	0.00	0.21	0.11	−0.05
Carbohydrate - sugars (g)	−0.26	−0.22	0.06	−0.06	0.26	0.02	0.01
Protein (g)	−0.13	0.13	0.19	0.26	0.08	−0.09	−0.02
Fat – total (g)	−0.31	0.02	0.00	0.10	−0.11	−0.01	0.17
MUFA (g)	−0.32	0.03	−0.06	0.08	−0.14	−0.01	0.16
PUFA (g)	−0.36	0.17	0.07	0.16	−0.15	−0.07	0.23
SFA (g)	−0.29	−0.03	0.05	0.04	−0.04	0.01	0.10
Cholesterol (mg)	−0.18	0.13	0.22	0.25	−0.03	−0.03	−0.18
Alcohol (g)	0.40	−0.07	−0.09	0.05	0.37	0.05	−0.11
Sodium (mg)	−0.21	0.03	0.20	0.33	0.11	0.02	−0.02
Chloride (mg)	−0.25	0.04	0.19	0.31	0.06	0.02	−0.03
Calcium (mg)	−0.17	0.09	0.17	0.17	0.09	−0.06	0.00
Phosphorus (mg)	−0.17	0.17	0.25	0.21	0.10	−0.06	−0.09

	FPG	Comet	MN	NBUD	NPB	NDI	Apoptotic cells
Magnesium (mg)	−0.13	0.05	0.11	0.15	0.11	−0.07	−0.03
Potassium (mg)	−0.06	−0.02	0.17	0.12	0.23	−0.07	−0.13
Iron (mg)	−0.14	−0.03	0.15	0.13	0.09	0.04	−0.16
Copper (mg)	−0.11	0.04	0.09	0.08	0.14	0.13	−0.07
Zinc (mg)	−0.12	0.06	0.17	0.14	−0.02	−0.02	−0.15
Selenium (µg)	−0.12	0.13	0.24	0.20	0.02	0.02	−0.21
Manganese(mg)	−0.37	−0.05	0.15	0.13	−0.02	−0.12	−0.06

FPG – formamidopyrimidine-DNA glycosylase; MN – micronuclei; NBUD – nuclear bud; NPB – nucleoplasmic bridge; NDI − nuclear division indeks; MUFA i PUFA − monounsaturated fatty acids and. polyunsaturated fatty acids; SFA - Saturated Fatty Acids; FFQ − Food Frequency Questionnaire

Supplementary Table S6. Spearman's correlation between average vitamin intake (FFQ) and DNA damage parameters in the SRD group with statistical significance ($p < 0.999$)

	FPG	Comet	MN	NBUD	NPB	NDI	Apoptotic cells
α carotene (μg)	−0.43	0.10	0.07	−0.02	−0.25	0.05	−0.15
β carotene (μg)	−0.39	0.10	0.11	0.17	0.05	−0.09	0.00
Vitamin A – retinol (μg)	0.04	0.14	0.07	0.07	−0.02	0.19	−0.01
Vitamin B2 – riboflavin (mg)	−0.05	0.27	0.25	0.24	0.21	−0.14	0.00
Vitamin B1 – thiamin (mg)	−0.01	0.10	0.14	0.15	0.26	−0.03	−0.06
Vitamin B12 – cobalamin (μg)	−0.04	0.42	0.11	0.14	0.00	0.01	−0.05
Total folate (μg)	−0.28	0.08	0.27	0.14	0.07	−0.05	−0.17
Niacin (mg)	0.04	0.19	−0.03	0.16	0.16	−0.10	0.01
Vitamin B6 – pyridoxine (mg)	−0.04	0.01	0.11	0.07	0.07	0.05	−0.25
Vitamin C - ascorbic acid (mg)	−0.28	0.05	0.10	0.14	0.19	−0.15	0.00

	FPG	Comet	MN	NBUD	NPB	NDI	Apoptotic cells
Vitamin D – ergocalciferol (μg)	0.05	0.16	−0.14	0.11	−0.03	−0.02	0.05
Vitamin E - alpha tocopherol (mg)	−0.22	0.06	0.06	0.08	0.04	−0.01	0.14

FPG – formamidopyrimidine-DNA glycosylase; MN – micronuclei; NBUD – nuclear bud; NPB – nucleoplasmic bridge; NDI − nuclear division indeks; FFQ − Food Frequency Questionnaire

Supplementary Table S7. Spearman's correlation coefficient (r) between the change in Dietary Inflammatory Index (ΔDII) and the change in DNA damage parameters (Δ=T1–T0) in the SRD and VLCD groups.

Variables		Δ DII	
		SRD	VLCD
ΔfreqMN	r	0.095	0.132
	p	0.636	0.539
	N	27	24
Δ freqNBUD	r	0.062	0.031
	p	0.757	0.887
	N	27	24
Δ MNNBUD	r	0.078	0.077
	p	0.699	0.719
	N	27	24
Δ NPB	r	-0.034	-0.242
	p	0.868	0.254
	N	27	24
Δ apoptotic cells	r	0.108	0.185
	p	0.591	0.387
	N	27	24
Δ necrotic cells	r	-0.076	-0.005
	p	0.708	0.983
	N	27	24
Δ Tail Intensity Mean	r	0.002	0.148
	p	0.990	0.491

Variables		Δ DII	
		SRD	VLCD
	N	27	24
Δ FPG	r	0.098	0.179
	p	0.628	0.402
	N	27	24
Lymphocytes	r	-0.174	-0.289
	p	0.385	0.171
	N	27	24
Lymphocytes %	r	-0.171	0.054
	p	0.394	0.804
	N	27	24
Glucose	r	-0.205	-0.189
	p	0.304	0.376
	N	27	24
Insulin	r	-0.034	0.120
	p	0.866	0.576
	N	27	24

Δ = T1- T0; bold $p<0.05$.
